# Maternal iron status in early pregnancy and birth outcomes: insights from the Baby's Vascular health and Iron in Pregnancy study

**DOI:** 10.1017/S0007114515001166

**Published:** 2015-05-06

**Authors:** Nisreen A. Alwan, Janet E. Cade, Harry J. McArdle, Darren C. Greenwood, Helen E. Hayes, Nigel A. B. Simpson

**Affiliations:** 1 Academic Unit of Primary Care and Population Sciences, Faculty of Medicine, University of Southampton, Southampton General Hospital, Southampton SO16 6YD, UK; 2 Nutritional Epidemiology Group, School of Food Science and Nutrition, University of Leeds, Leeds LS2 9JT, UK; 3 Rowett Institute of Nutrition and Health, University of Aberdeen, Aberdeen, UK; 4 Division of Biostatistics, University of Leeds, Leeds, UK; 5 Division of Women's and Children's Health, University of Leeds, Leeds, UK

**Keywords:** Iron, Birth weight, Preterm birth, Pregnancy

## Abstract

Fe deficiency anaemia during early pregnancy has been linked with low birth weight and preterm birth. However, this evidence comes mostly from studies measuring Hb levels rather than specific measures of Fe deficiency. The present study aimed to examine the association between maternal Fe status during the first trimester of pregnancy, as assessed by serum ferritin, transferrin receptor and their ratio, with size at birth and preterm birth. In the Baby VIP (Baby's Vascular health and Iron in Pregnancy) study, we recruited 362 infants and their mothers after delivery in Leeds, UK. Biomarkers were measured in maternal serum samples previously obtained in the first trimester of pregnancy. The cohort included sixty-four (18 %) small for gestational age (SGA) babies. Thirty-three babies were born preterm (9 %; between 34 and 37 weeks). First trimester maternal Fe depletion was associated with a higher risk of SGA (adjusted OR 2·2, 95 % CI 1·1, 4·1). This relationship was attenuated when including early pregnancy Hb in the model, suggesting it as a mediator (adjusted OR 1·6, 95 % CI 0·8, 3·2). For every 10 g/l increase in maternal Hb level in the first half of pregnancy the risk of SGA was reduced by 30 % (adjusted 95 % CI 0, 40 %); levels below 110 g/l were associated with a 3-fold increase in the risk of SGA (95 % CI 1·0, 9·0). There was no evidence of association between maternal Fe depletion and preterm birth (adjusted OR 1·5, 95 % 0·6, 3·8). The present study shows that depleted Fe stores in early pregnancy are associated with higher risk of SGA.

Fe deficiency (ID) is the leading single nutrient deficiency in the world. The WHO considers it a public health condition of epidemic proportions with significant consequences including loss of earnings, general ill-health and premature death^(^ ^1^ ^)^. Anaemia, a common outcome of ID, is therefore unsurprisingly prevalent in expectant mothers, affecting nearly half of all pregnant women worldwide^(^ ^2^ ^)^. Maternal Fe deficiency anaemia (IDA) has been linked to higher risk of low birth weight, preterm delivery and infant IDA, which in turn can permanently impair intelligence, motor and behavioural development, and increase risk of future IDA in the offspring^(^ ^3^ ^–^ ^12^ ^)^.

There is a stepwise progression towards IDA: initial depletion of Fe stores, followed by Fe deficient erythropoiesis (IDE), then reduction in Hb concentration^(^ ^13^ ^)^. Anaemia therefore represents the final stage of ID; it occurs as tissue and cellular stores are progressively exhausted; its manifestation is most readily ascertained through assessment of Hb levels. However, non-anaemic ID as a separate pathological entity is not well recognised, and its association with birth outcomes is inadequately investigated. The prevalence of ID during pregnancy (low serum ferritin (sF) and sparse or absent stainable Fe in bone marrow) is probably far greater than the prevalence of anaemia^(^ ^4^ ^)^. ID, without anaemia, has been associated with reduced exercise capacity, impaired temperature regulation and impaired cognitive function in animal and human studies^(^ ^14^ ^)^, and may therefore implicate risks for mother and baby in pregnancy. Studies assessing ID status require further measurements other than Hb concentrations.

The biomarker sF is the most widely used one in this respect. It is an indicator of body storage Fe. In women, levels under 15 μg/l indicate depleted Fe stores^(^ ^15^ ^)^. However, it is affected by inflammatory conditions and therefore may not be specific to distinguishing ID. Levels between 12 and 100 μg/l are difficult to interpret because inflammation can cause elevation of sF, even in the presence of ID^(^ ^16^ ^)^.

Circulating serum transferrin receptor (sTfR) is a soluble form of the membrane receptor produced by proteolytic cleavage. Both the expression of transferrin receptor on the cell surface and the concentration of the soluble transferrin receptor are inversely related to the level of intracellular Fe^(^ ^17^ ^)^. Measuring sTfR may provide more specific information, and it has the advantage over sF in that it can distinguish IDA from anaemia of chronic inflammation, as well as identify Fe depletion and functional ID in patients with concurrent inflammation^(^ ^18^ ^)^. The ratio of sTfR:sF ratio is considered the gold standard marker of Fe status^(^ ^19^ ^)^, and has been used to assess Fe status in pregnant populations such as in the United States National Health and Nutrition Examination Survey^(^ ^20^ ^)^.

IDE is the most common cause of raised sTfR. Depleted Fe stores, without IDE, is not associated with raised sTfR, and is best indicated by low sF concentration. As ID progresses beyond depletion of Fe stores into negative Fe status, with inadequate Fe supply from erythropoiesis, sTfR levels begin to rise, and continue to rise as IDE progressively worsens before the development of anaemia. The sTfR:sF ratio quantifies the entire spectrum of Fe status from positive Fe stores through negative Fe balance, and is particularly useful in evaluating Fe status in population studies^(^ ^13^ ^)^. A close linear relationship is demonstrable between the logarithm of the concentrations in μg/l, of sTfR:sF ratio with body Fe as calculated from the Hb Fe after correction for the absorption of dietary Fe^(^ ^21^ ^)^.

The potential consequences of ID in pregnancy are significant. Being born small for gestational age (SGA) or preterm are both associated with short- and long-term adverse health consequences including an increased risk of infant mortality, as well as increased risk of cognitive dysfunction and CVD later in life^(^ ^26^ ^)^.

The present study therefore aimed to examine the relationship between maternal Fe status assessed in early pregnancy with size at birth and gestational age, and to investigate whether maternal Hb level is a mediator in this relationship.

## Methods

### Study design

The Baby VIP (Baby's Vascular health and Iron in Pregnancy) study is a historical cohort study. Women aged 18 years or over who gave birth to live offspring at the Leeds Teaching Hospitals Trust maternity unit at a gestational age of 34 weeks or over, and had sufficient proficiency in English to understand what is involved in study participation, give consent and complete a written questionnaire were eligible to be included in the study. Study exclusion criteria included stillbirth or neonatal death, serious maternal illness, and babies with concurrent CVD (such as patent ductus arteriosus), inherited disorders of metabolism or congenital malformations. Participants were approached for the study on the postnatal wards after delivery. Each mother was asked permission to access hers and her baby's medical notes. The study was conducted between February 2012 and January 2013. Ethical approval was obtained from the South Yorkshire Research Ethics Committee of the NHS National Research Ethics Service (reference no. 11/YH/0064). All procedures were in accordance with the Helsinki Declaration of 1975 as revised in 1983. Participants provided their written informed consent to participate in the present study.

### Outcome measurement

Birth weight (in g) and gestational age (in d) were ascertained from the hospital delivery records. Customised birth weight centiles were calculated taking into account gestational age, maternal height, maternal pre-pregnancy or booking weight, ethnicity, parity and neonatal sex^(^ ^27^ ^)^. SGA was defined as less than the tenth customised birth weight centile. Duration of gestation was calculated from the date of the last menstrual period, and confirmed by ultrasound scans dating at about 12 and 20 weeks gestation. Preterm birth was defined as less than 37+0 weeks gestation.

### Exposure measurement

Maternal serum samples previously acquired and stored during the first trimester of pregnancy as part of routine antenatal care were identified and then analysed. The biomarker sF was measured using ELISA (Demeditec Diagnostics GmbH) following the manufacturer's instructions. Briefly, 10****μl of plasma was treated in a sandwich ELISA method, using fluorometric measurements and calibrated, using standards supplied by the manufacturer. Quality controls were included as appropriate. Data were expressed in μg/l. The WHO's cut-off of 15****μg/l in sF was used to indicate depleted Fe stores^(^ ^15^ ^)^. And sTfR assays were performed using a commercially available kit, based on a polyclonal antibody in a sandwich enzyme immunoassay format (DTFR1; R&D Systems). This yielded sTfR levels in nmol/l units. The values were converted to μg/l using a molecular weight of sTfR of 75****000****Da (R&D technical data sheet). The sTfR:sF ratio was obtained by dividing sTfR over sF (μg/l:μg/l). This was logged to obtain a normal distribution.

In the UK, Hb is measured routinely in pregnancy at about 12 and 28 weeks gestation. Maternal Hb values were extracted from the antenatal care records and/or the hospital electronic results server. A cut-off of 110****g/l in Hb was used to indicate anaemia at 20 weeks gestation or less, and 105****g/l to indicate anaemia beyond 20 weeks gestation, following the National Institute for Health and Care Excellence guidelines^(^ ^28^ ^)^.

### Covariable assessment

A basic demographic and lifestyle questionnaire was administered to the mother by the research team at the time of recruitment. The Index of Multiple Deprivation (IMD) was derived from the participants' postcodes, using the GeoConvert tool which utilises the 2001 UK census data (http://geoconvert.mimas.ac.uk). Parity, maternal height, weight, ethnicity, smoking, pregnancy complications (pre-eclampsia, gestational diabetes), blood pressure measurements and intake of Fe supplements were extracted from the mother and the baby's clinical records.

### Statistical methods

Statistical analysis was performed using Stata version 11 (StataCorp, 2009). Univariable analysis was performed using independent sample *t* test, one-way ANOVA or Mann-Whitney test for continuous variables, and χ^2^ test for categorical variables. Multivariable linear regression was performed with customised birth weight centile and gestational age as the main outcomes, and indicators of maternal Fe status as predictors. Multiple logistic regression was performed with SGA and preterm birth as binary outcomes. The models with gestational age/preterm birth as outcomes were adjusted for maternal covariables including age, BMI, parity, ethnicity, smoking status, the presence of gestational diabetes or pre-eclampsia, blood pressure at booking and 36 weeks gestation, and IMD score. Sensitivity analyses were performed taking into account the intake of Fe supplements during pregnancy. Models with customised birth weight centile/SGA as outcomes were adjusted for the variables listed above excluding maternal ethnicity, parity and BMI which were taken into account in the centile calculation.

Hb levels in early ( ≤ 20 weeks) and late (>20 weeks) pregnancy were investigated as a mediator in the relationship between maternal Fe status as expressed by sF and sTfR:sF ratio, and birth outcomes, by adjusting for it in the relevant models. If an association was attenuated, this could indicate the presence of mediation^(^ ^29^ ^)^.

For a difference in customised birth weight centile between Fe-deficient and non-Fe deficient mothers of 10 centile points, using a mean of 45 centile points, a standard deviation of 29^(^ ^30^ ^,^ ^31^ ^)^, and a prevalence of ID of 25 %^(^ ^32^ ^,^ ^33^ ^)^, a sample size of 356 mother–baby pairs was required to achieve *P =*0·05 with 80 % power.

## Results

In total, 362 women living in Leeds, the UK and surrounding areas were recruited for the present study. Of them 288 (80 %) were of white ethnic origin, thirty-seven (10 %) of Asian origin, thirteen (4 %) of African-Caribbean origin, eleven (3 %) of mixed origin and thirteen (4 %) of other ethnic origin. Mean maternal age was 31 (sd 6) years, and mean maternal pre-pregnancy BMI was 26 (sd 6) kg/m^2^. Among them 173 women were primiparous (40 %), and half of the babies were male. Mean birth weight was 3329 (sd 632) g, with forty (11 %) babies weighing less than 2500 g, and 358 women had sufficient information to derive customised birth weight centiles. Mean customised birth weight centile was 41 (sd 29), with sixty-four (18 %) babies weighing less than the 10th centile (SGA), and twenty-nine (8 %) babies weighing less than the 3rd centile. Twenty (6 %) babies weighed more than the 90th centile. Mean gestational age was 277 (sd 14) d i.e. 39·6 weeks. Thirty-three (9 %) babies were born preterm (all between 34 and 37 weeks gestation).

Out of 348 pregnant women with information on sF in the first trimester, seventy-nine (23 %) had Fe depletion according to the WHO cut-off of 15 μg/l. This proportion was 46 % in women of Asian origin compared to 20 % in women of white origin. Median sF was 13·7 μg/l (interquartile range 16·9–62·4). Mean maternal Hb was 126 g/l and 116 g/l (sd 10) in the first and second halves of pregnancy respectively. Prevalence of anaemia at ≤ 20 weeks (Hb < 110 g/l) and >20 weeks gestation (Hb < 105 g/l) was 5 % (16/329) and 14 % (48/337) respectively. Only half of anaemic women in the first half (*n* 8), and 45 % of anaemic women in the second half of pregnancy (*n* 22), had a first trimester sF of less than 15 μg/l. However, fourteen (89 %) of anaemic women in the first half of pregnancy and forty-three (90 %) of anaemic women in the second half of pregnancy had sF of less than 70 μg/l.

Also 121 women (34 %) took Fe supplements at some stage during pregnancy. Eight (2 %) started taking them in the first trimester, compared to sixty-seven (18·6 %) who did so in the second trimester, and forty-six (13 %) in the third trimester. Out of those with Fe depletion in the first trimester (sF < 15 μg/l), only forty-six (58 %) had Fe supplements during their pregnancy, compared to thirteen (81 %) of anaemic women in the first half of pregnancy, and forty (83 %) of anaemic women in the second half of pregnancy.

Women with SGA babies were more likely to smoke, have lower early pregnancy Hb, be anaemic at ≤ 20 weeks gestation, have Fe depletion in the first trimester (sF < 15 μg/l) and have suffered from pre-eclampsia during pregnancy, compared to women with babies who were not SGA. In women with Fe depletion in the first trimester of pregnancy, 25 % had SGA babies compared with 14 % of women with no Fe depletion. Table 1 describes the characteristics of participants whose babies were born SGA compared to those with babies who were not SGA.

**Table 1:** Characteristics of Baby's Vascular health and Iron in Pregnancy study participants (*n* 362) by size at birth (Mean values and standard deviations; median values and interquartile ranges (IQR); percentages and 95 % confidence intervals)

	SGA (*n* 64)*	Not SGA (*n* 294)	
	Mean	sd	Mean	sd	*P* †
Maternal age at antenatal booking (years)	30·4	6·0	30·5	5·7	0·8
Maternal BMI at antenatal booking (kg/m^2^)	25·7	5·8	26·3	5·8	0·4
Maternal Hb at ≤ 20 weeks gestation (g/l)	123	11	127	10	0·02
Maternal Hb at >20 weeks gestation (g/l)	116	11	116	10	0·9
IMD	30·6	19·2	29·2	19·8	0·6
Gestational age (d)	270	15	279	13	< 0·0001
Birth weight (g)	2499	446	3510	516	< 0·0001
First trimester maternal sF (μg/l)					0·8
Median	33·5	30·7	
IQR	12·4–52·5	17·9–65·7	
First trimester maternal sTfR (nmol/l)					0·1
Median	13·6	12·5	
IQR	10·0–17·9	10·3–16·0	
First trimester maternal sF:sTfR ratio (μg/l)					0·6
Median	38·1	28·0	
IQR	16·4–101·2	14·4–61·2	
	%	95 % CI	%	95 % CI	
Primiparous	41	29, 54	49	44, 55	0·2
Maternal smoking at antenatal booking	22	13, 35	12	9, 17	0·04
Maternal White ethnicity	75	63, 85	81	76, 85	0·3
Maternal Asian ethnicity	16	8, 27	9	6, 13	-
Maternal African Caribbean ethnicity	3	0, 11	3	1, 6	-
Anaemia at ≤ 20 weeks gestation ( < 110 g/l)	11	4, 23	4	2, 7	0·02
Anaemia at >20 weeks gestation ( < 105 g/l)	15	6, 27	14	10, 19	0·9
Gestational diabetes	3	0, 11	1	0, 3	0·3
Pre-eclampsia	5	1, 13	1	0, 3	0·04
Male baby	45	33, 58	51	46, 57	0·4
Preterm birth (34–37 weeks gestation)	19	10, 31	7	5, 11	0·004

SGA, small for gestational age; IMD, Index of Multiple Deprivation; sF, serum ferritin; sTfR, serum transferrin receptor.

* < 10th birth weight centile.

† Independent samples *t* test or Mann-Whitney test for continuous variables, and χ^2^ test for categorical variables.

There was no evidence of an association between maternal sF, sTfR, log sTfR:sF ratio with customised birth weight centile. In univariable analysis, maternal anaemia in early pregnancy was associated with reduction of 15 centile points in birth weight (95 % CI 1, 29; *P*= 0·04). However, this association was attenuated when adjusting for maternal age, smoking, gestational diabetes, pre-eclampsia and IMD (adjusted difference in customised birth weight centile = -11 centile points, 95 % CI -25, 3; *P*= 0·1) (Table 2).

**Table 2:** Associations of customised birth weight centile with indicators of iron status during pregnancy in the Baby's Vascular health and Iron in Pregnancy study (Differences and 95 % confidence intervals)

	Difference in birth weight centile mean*
Predictor	Unadjusted difference	95 % CI for unadjusted difference	*P*	Adjusted difference†	95 % CI for adjusted difference	*P*	*n* (multivariable model)
Maternal sF at 12 weeks gestation (per 10 μg/l change)	− 0·1	− 1·0, 0·6	0·9	0·1	− 0·6, 0·7	0·9	341
Maternal Fe depletion at 12 weeks gestation (sF < 15 μg/l)	− 4·2	− 11·3, 2·3	0·3	− 4·6	− 11·7, 2·5	0·2	341
Maternal sTfR at 12 weeks gestation (per nmol/l)	− 0·2	− 0·7, 0·4	0·5	− 0·3	− 0·8, 0·3	0·3	341
Maternal log sTfR:sF ratio at 12 weeks gestation (per μg/l)	− 0·8	− 3·4, 1·7	0·5	− 1·2	− 3·8, 1·3	0·3	341
Maternal Hb at ≤ 20 weeks gestation (per 10 g/l)	1·4	− 1·7, 4·5	0·4	1·0	− 2·0, 4·1	0·6	326
Maternal Hb at >20 weeks gestation (per 10 g/l)	− 0·8	− 3·7, 2·2	0·6	− 1·2	− 4·4, 1·4	0·3	334
Maternal anaemia at ≤ 20 weeks gestation ( < 110 g/l)	− 15·0	− 29·2, − 1·0	0·04	− 11·2	− 25·4, 3·1	0·1	326
Maternal anaemia at >20 weeks gestation ( < 105 g/l)	− 4·5	− 13·1, 4·2	0·3	− 1·4	− 10·1, 7·3	0·8	334

sF, serum ferritin; sTfR, serum transferrin receptor.

* Customised birth weight centile (takes into account maternal pre-pregnancy weight, height, ethnicity, parity, gestational age and baby's sex).

† Adjusted for maternal age, smoking, gestational diabetes, pre-eclampsia and area deprivation score (Index of Multiple Deprivation).

Maternal Fe depletion in the first trimester (sF < 15 μg/l) was associated with a higher risk of a SGA baby (adjusted OR 2·2, 95 % CI 1·1, 4·1; *P*= 0·02). Adjusting for maternal Fe supplement intake in a sensitivity analysis did not alter this association (adjusted OR 2·3, 95 % CI 1·2, 4·5; *P*= 0·02). However, this relationship was attenuated when including early pregnancy Hb in the model (adjusted OR 1·6, 95 % CI 0·8, 3·2; *P*= 0·2). For every 10 g/l increase in maternal Hb level in the first half of pregnancy the risk of SGA was reduced by 30 % (adjusted 95 % CI 0, 40 %; *P*= 0·03), with levels < 110 g/l associated with 3-fold increase in the risk of SGA (95 % CI 1·0, 9·0; *P*= 0·05). Maternal sTfR was also associated in the multivariable model adjusting for maternal age, smoking, gestational diabetes, pre-eclampsia and IMD with SGA (adjusted OR 1·1 for every 1 nmol/l increase in sTfR, 95 % CI 1·0,1·1; *P*= 0·04). However, there was no evidence of association of SGA with maternal sTfR:sF ratio (Table 3).

**Table 3:** Associations of being born small for gestational age with indicators of iron status during pregnancy in the Baby's Vascular health and Iron in Pregnancy study (Odds ratios and 95 % confidence intervals)

	OR of SGA*
Predictor	Unadjusted OR	95 % CI for unadjusted OR	*P*	Adjusted OR†	95 % CI for adjusted OR	*P*	*n* (multivariable model)
Maternal sF at 12 weeks gestation (μg/l)	1·0	0·9, 1·0	0·8	1·0	0·9, 1·0	0·7	341
Maternal Fe depletion at 12 weeks gestation (sF < 15 μg/l)	2·0	1·1, 3·7	0·02	2·2	1·1, 4·1	0·02	341
Maternal sTfR at 12 weeks gestation (nmol/l)	1·0	1·0, 1·1	0·1	1·1	1·0, 1·1	0·04	341
Maternal log sTfR:sF ratio at 12 weeks gestation (μg/l)	1·1	0·9, 1·4	0·3	1·2	0·9, 1·5	0·1	341
Maternal Hb at ≤ 20 weeks gestation (per 10 g/l)	0·7	0·6, 1·0	0·03	0·7	0·6, 1·0	0·03	326
Maternal Hb at >20 weeks gestation (per 10 g/l)	1·0	0·7, 1·3	0·9	1·1	0·8, 1·4	0·7	334
Maternal anaemia at ≤ 20 weeks gestation ( < 110 g/l)	3·3	1·1, 9·4	0·03	3·0	1·0, 9·0	0·05	326
Maternal anaemia at >20 weeks gestation ( < 105 g/l)	1·0	0·5, 2·3	0·9	0·8	0·4, 2·0	0·7	334

SGA, small for gestational age; sF, serum ferritin; sTfR, serum transferrin receptor.

* < 10th customised birth weight centile (takes into account maternal pre-pregnancy weight, height, ethnicity, parity, gestational age and baby's sex).

† Adjusted for maternal age, smoking, gestational diabetes, pre-eclampsia, and area deprivation score (Index of Multiple Deprivation).

There was no evidence of association between maternal Fe status measured by sF, sTfR or log sTfR:sF ratio with preterm birth or gestational age. However, there was an association observed between early pregnancy maternal Hb and gestational age in univariable analysis. For every 10 g/l increase in early pregnancy Hb, there was an increase in gestational age by 2 d (95 % CI 0·2, 3·0; *P*= 0·03). This association was attenuated in the multivariable model adjusting for maternal age, ethnicity, parity, pre-pregnancy BMI, smoking, gestational diabetes, pre-eclampsia and IMD score. Mothers who were anaemic in the first half of pregnancy had on average a gestation shorter by 7 d (adjusted 95 % CI 0, 14; *P*= 0·05) compared to non-anaemic mothers (Tables 4 and 5).

**Table 4:** Associations of gestational age with indicators of iron status during pregnancy in the Baby's Vascular health and Iron in Pregnancy study (Differences and 95 % confidence intervals)

	Difference in gestational age mean (d)
Predictor	Unadjusted difference	95 % CI for unadjusted difference	*P*	Adjusted difference*	95 % CI for adjusted difference	*P*	*n* (multivariable model)
Maternal sF at 12 weeks gestation (per 10 μg/l change)	0·1	− 0·2, 0·4	0·6	− 0·2	− 0·4, 0·2	0·6	341
Maternal Fe depletion at 12 weeks gestation (sF < 15 μg/l)	0·3	− 3·4, 4·0	0·9	0·02	− 3·4, 3·4	0·9	341
Maternal sTfR at 12 weeks gestation (nmol/l)	− 0·1	− 0·2, 0·2	0·5	− 0·1	− 0·3, 0·2	0·5	341
Maternal log sTfR:sF ratio at 12 weeks gestation (μg/l)	− 0·6	− 1·8, 0·6	0·3	0·01	− 1·2, 1·3	0·9	341
Maternal Hb at ≤ 20 weeks gestation (per 10 g/l)	1·6	0·2, 3·0	0·03	1·1	− 0·4, 2·6	0·1	326
Maternal Hb at >20 weeks gestation (per 10 g/l)	0·1	− 1·3, 1·5	0·9	− 0·5	− 1·9, 0·9	0·5	334
Maternal anaemia at ≤ 20 weeks gestation ( < 110 g/l)	− 9·1	− 15·8, − 2·4	0·008	− 6·8	− 13·6, 0·1	0·05	326
Maternal anaemia at >20 weeks gestation ( < 105 g/l)	1·6	− 2·4, 5·6	0·4	3·7	− 0·4, 7·8	0·07	334

sF, serum ferritin; sTfR, serum transferrin receptor.

* Adjusted for maternal age, pre-pregnancy BMI, ethnicity, parity, smoking, gestational diabetes, pre-eclampsia, and area deprivation score (Index of Multiple Deprivation).

**Table 5:** Associations of being born preterm with indicators of iron status during pregnancy in the Baby's Vascular health and Iron in Pregnancy study (Odds ratios and 95 % confidence intervals)

	OR of preterm birth ( < 37 weeks gestation)
Predictor	Unadjusted OR	95 % CI of unadjusted OR	*P*	Adjusted OR*	95 % CI of adjusted OR	*P*	*n* (multivariable model)
Maternal sF at 12 weeks gestation (μg/l)	1·0	0·9, 1·0	0·6	1·0	0·9, 1·0	0·6	341
Maternal Fe depletion at 12 weeks gestation (sF < 15 μg/l)	1·6	0·7, 3·7	0·3	1·5	0·6, 3·8	0·4	341
Maternal sTfR at 12 weeks gestation (nmol/l)	1·0	1·0, 1·1	0·5	1·0	1·0, 1·1	0·5	341
Maternal log sTfR:sF ratio at 12 weeks gestation (μg/l)	1·1	0·8, 1·4	0·8	1·0	0·7, 1·5	0·8	341
Maternal Hb at ≤ 20 weeks gestation (per 10 g/l)	0·8	0·6, 1·1	0·2	0·9	0·6, 1·4	0·7	326
Maternal Hb at >20 weeks gestation (per 10 g/l)	1·2	0·8, 1·7	0·5	1·3	0·9, 1·9	0·2	334
Maternal anaemia at ≤ 20 weeks gestation ( < 110 g/l)	2·6	0·7, 9·5	0·2	1·3	0·3, 6·2	0·8	326
Maternal anaemia at >20 weeks gestation ( < 105 g/l)	0·4	0·1, 1,9	0·3	0·2	0·04, 1·0	0·05	334

sF, serum ferritin; sTfR, serum transferrin receptor.

* Adjusted for maternal age, pre-pregnancy BMI, ethnicity, parity, smoking, gestational diabetes, pre-eclampsia, and area deprivation score (Index of Multiple Deprivation).

## Discussion

The present study in an inner city population sample of women, with about 20 % from ethnic minority background, shows that maternal Fe depletion in early pregnancy was associated with a greater odd of an SGA birth. This relationship appeared to be mediated by early pregnancy maternal Hb, which was independently and inversely associated with SGA.

### Interpretation of results

In the present study, anaemia in the first half of pregnancy was associated with a higher risk of having a baby who is SGA, and every 10 g/l increase in early pregnancy maternal Hb (before 20 weeks gestation) was associated with a 30 % reduction in the risk of SGA. This result supports the previous evidence of association between early maternal Hb and anaemia with the risk of low birth weight^(^ ^34^ ^,^ ^35^ ^)^.

As it was previously demonstrated^(^ ^36^ ^)^, maternal Hb in the second half of pregnancy was not associated with SGA in the present study as well. In contrast, a previous study from our group, conducted 10 years earlier, found a 40 % increase in the risk of SGA with every 10 g/l increase in maternal Hb at 28 weeks gestation in a sample size of 572 women^(^ ^37^ ^)^. Although the proportion of participants with late pregnancy anaemia in that study (23 %) was higher than the proportion observed in the present study (14 %), mean Hb in both studies was similar. Why the results are different is not obvious, but may be related to the time course of plasma volume expansion in the different populations.

There was no evidence of association between the incidence of preterm birth and early pregnancy maternal Hb or anaemia in the present study contrary to the findings of previous studies^(^ ^38^ ^–^ ^40^ ^)^. However, maternal anaemia in the first half of pregnancy was marginally associated with a reduction in gestational age, when analysed as a continuous outcome, while maternal anaemia in the second half of pregnancy was marginally associated with a reduction in the risk of preterm birth.

The evidence available in the literature of the association between maternal anaemia and birth outcomes suggests that it is U-shaped^(^ ^41^ ^)^. Causes of adverse birth outcomes may differ at the extremes of the maternal Hb range. While low Hb in early pregnancy may reflect ID or other nutritional deficiencies such as vitamin B or folic acid, high Hb values later in pregnancy may reflect inadequate expansion of plasma volume. Rasmussen^(^ ^41^ ^)^ suggests that this U-shaped association is spurious due to the design of research evidence available; it is more apparent in studies that use ‘lowest Hb’ than in those that control for the stage of gestation, or include data only from women very early in pregnancy, when changes in plasma volume are minimal^(^ ^11^ ^,^ ^38^ ^)^.

Participants in the present study who were Fe-depleted at the beginning of pregnancy were twice as likely to have SGA babies, compared to those who were Fe-replete. Maternal sTfR level measured in the first trimester of pregnancy (which increases in ID) was also marginally associated with higher risk of SGA. These results are consistent with findings from previous studies^(^ ^42^ ^)^.

The relationship between maternal Fe depletion in the first trimester and SGA was tested for mediation by maternal Hb, as it was an independent predictor of SGA. Including maternal Hb in the model attenuated the relationship between maternal Fe depletion and SGA. This may point to the possibility that the mechanism through which inadequate body Fe could potentially result in small size at birth is through the efficiency of carrying oxygen to the placenta, which is reduced by a reduction in maternal Hb concentrations. In animal models IDA also increases oxidative stress levels in the liver, heart, kidney and placenta, as well as hypoxia and inflammation in placenta^(^ ^43^ ^)^. On the other hand, there could be another pathway of association between anaemia in pregnancy and adverse offspring outcomes other than ID, such as other nutrient deficiencies.

A major problem in interpreting Hb values and how they relate to maternal Fe status is the physiological process of plasma volume expansion in pregnancy. This leads to a fall in Hb level which obscures the typical relationship between Fe status and Hb levels. Not only that, it also makes it difficult to interpret plasma-based indicators of Fe depletion including sF due to plasma dilution. Also, the point at which Hb is assessed is of utmost importance, as plasma volume and cell mass change in the different stages of pregnancy^(^ ^41^ ^)^. Therefore, assessing Hb in early pregnancy, before the materialisation of plasma expansion, reflects Fe status better than later in pregnancy.

### Strengths and limitations

Birth weight was ascertained objectively from the medical birth records. Gestational age was calculated using information from a dating ultrasound scan at the end of the first trimester of pregnancy and extracted from the medical records. Therefore, these two outcome measures were not subject to measurement bias. We have adjusted the main study outcome for maternal weight, height, ethnicity, parity as well as baby's sex and gestational age using the GROW-centile calculator, which has been recommended by the UK Royal College of Obstetrics and Gynaecology for assessment of birth weight^(^ ^44^ ^)^. We have also adjusted the statistical models for a measure of deprivation, the IMD, however, it is worth noting that this is an area-based rather than an individual-based measure.

The present study assessed the exposure of interest prospectively, as the maternal serum samples were collected in the first trimester of pregnancy. Information on maternal Hb and Fe supplements was ascertained objectively from the medical records, rather than by self-reporting. The best available measure, which utilised the ferritin and transferrin receptor biomarkers in a ratio that relates directly to total body Fe stores, was used to assess maternal Fe status^(^ ^19^ ^)^. A study set out to determine the diagnostic value of sTfR:sF ratio to determine body Fe stores against bone marrow aspirate examination showed that sTfR:sF ratio had the best diagnostic efficiency with the sensitivity of 81 % and a specificity of 97 %. And sF alone, with a cut-off of 60 μg/l, had the same specificity but lower sensitivity (76 %)^(^ ^45^ ^)^.

Deriving body Fe stores estimates from sTfR:sF ratio is limited by the current availability of several commercial assays that yield different sTfR values. The calculation formula provided by Cook^(^ ^46^ ^)^ used to deduct body Fe stores values can only be used if sTfR assay commutability is established. Furthermore, the calculation is complicated by different inflammatory responses in pregnancy, which may affect ferritin values. There is a pressing need to calibrate sTfR assays against international reference standards to provide comparability across studies.

### Implications for research and practice

Currently in the UK, routine Fe supplementation is not recommended in pregnancy. Pregnant women are only screened for anaemia which is at the extreme end of ID spectrum. Screening for ID in women at an early stage in pregnancy may be beneficial, since it has been shown in the present study and other research that it may predict adverse birth outcomes including SGA. Correcting ID may mitigate or prevent this outcome. The ideal way to ascertain ID would be to test sF levels in the first trimester of pregnancy, preferably at the first antenatal booking visit. If sF was low, indicating ID based on international cut-off values, then Fe supplementation may be recommended plus dietary advice to increase intake from diet and maximise absorption of both non-haem Fe and supplements. The issue of the side effects of supplements, and optimisation of their absorption must be addressed with women who are deemed in need of them.

Research is needed to assess the cost-effectiveness of such a routine testing and selective supplementation approach on a population level, taking into account country-specific prevalence rates of SGA and ID in women of childbearing age.

Until that time, as part of routine antenatal care, emphasis should be on receiving more detailed and helpful dietary advice and ways to optimise nutritional status in pregnancy, including Fe intake and absorption from the diet. This could be delivered in a personalised fashion as part of the antenatal care package rather than mere signposting to leaflets and information websites. Incorporating dietary advice and follow-up in routine antenatal care is likely to have cost implications. Therefore, careful evaluation of the clinical and cost-effectiveness of such a personalised approach should be planned and conducted.

We are sincerely grateful to all the mothers who took part in the Baby VIP study. Our sincere thanks go to Julie Grindey, Angela Wray and Viv Dolby for data collection, Tony Evans and Ruth Owen for facilitating the processing of laboratory samples, and Christine Kennedy for advice on laboratory analysis.

## References

[1] WHO (2010) Micronutrient deficiencies http://www.who.int/nutrition/topics/ida/en/index.html (accessed accessed August 2014).

[2] UNICEF/UNU/WHO (2001) Iron Deficiency Anemia: Assessment, Prevention, and Control. Geneva: World Health Organization.

[3] Beard JL (2008) Why iron deficiency is important in infant development. J Nutr 138, 2534–2536. 19022985 10.1093/jn/138.12.2534 PMC3415871

[4] Allen L (2000) Anemia and iron deficiency: effects on pregnancy outcome. Am J Clin Nutr 71, Suppl., 1280S–1284S. 10799402 10.1093/ajcn/71.5.1280s

[5] Barbin B, Ginny M, Sapau J, et al. (1990) Consequences of maternal anaemia on outcome of pregnancy in a malaria endemic area in Papua New Guinea. Ann Trop Med Parasitol 84, 11–24. 2184786 10.1080/00034983.1990.11812429

[6] Bondevik G, Lie R, Ulstein M, et al. (2001) Maternal hematological status and risk of low birth weight and preterm delivery in Nepal. Acta Obstet Gynecol Scand 80, 402–408. 11328215

[7] Colomer J, Colomer C, Gutierrez D, et al. (1990) Anaemia during pregnancy as a risk factor for infant iron deficiency: report from the Valencia Infant Anaemia Cohort (VIAC) study. Paediatr Perinat Epidemiol 4, 196–204. 2362876 10.1111/j.1365-3016.1990.tb00638.x

[8] Godfrey K, Redman C, Barker D, et al. (1991) The effect of maternal anaemia and iron deficiency on the ratio of fetal weight to placental weight. Br J Obstet Gynaecol 98, 886–891. 1911607 10.1111/j.1471-0528.1991.tb13510.x

[9] Lone F, Qureshi R & Emanuel F (2004) Maternal anaemia and its impact on perinatal outcome. Trop Med Int Health 9, 486–490. 15078267 10.1111/j.1365-3156.2004.01222.x

[10] Scholl TO (2005) Iron status during pregnancy: setting the stage for mother and infant. Am J Clin Nutr 81, 1218S–1222S. 15883455 10.1093/ajcn/81.5.1218

[11] Zhou L-M, Yang W-W, Hua J-Z, et al. (1998) Relation of hemoglobin measured at different times in pregnancy to preterm birth and low birth weight in Shanghai, China. Am J Epidemiol 148, 998–1006. 9829872 10.1093/oxfordjournals.aje.a009577

[12] Kilbride J, Baker TG, Parapia LA, et al. (1999) Anaemia during pregnancy as a risk factor for iron-deficiency anaemia in infancy: a case–control study in Jordan. Int J Epidemiol 28, 461–468. 10405849 10.1093/ije/28.3.461

[13] Skikne BS (2008) Serum transferrin receptor. Am J Hematol 83, 872–875. 18821709 10.1002/ajh.21279

[14] McMahon LP (2010) Iron deficiency in pregnancy. Obstet Med 3, 17–24. 10.1258/om.2010.100004 PMC4989769 27582835

[15] World Health Organization (2011) Serum Ferritin Concentrations for the Assessment of Iron Status and Iron Deficiency in Populations. Geneva: WHO.

[16] Worwood M (1997) The laboratory assessment of iron status – an update. Clin Chim Acta 259, 3–23. 9086290 10.1016/s0009-8981(96)06488-1

[17] Baynes RD (1996) Assessment of iron status. Clin Biochem 29, 209–215. 8740506 10.1016/0009-9120(96)00010-k

[18] Allen J, Backstrom KR, Cooper JA, et al. (1998) Measurement of soluble transferrin receptor in serum of healthy adults. Clin Chem 44, 35–39. 9550555

[19] Zimmermann M (2008) Methods to assess iron and iodine status. Br J Nutr 99, S2–S9. 18598585 10.1017/S000711450800679X

[20] Mei Z, Cogswell ME, Looker AC, et al. (2011) Assessment of iron status in US pregnant women from the National Health and Nutrition Examination Survey (NHANES), 1999–2006. Am J Clin Nutr 93, 1312–1320. 21430118 10.3945/ajcn.110.007195

[21] Cook JD, Flowers CH & Skikne BS (2003) The quantitative assessment of body iron. Blood 101, 3359–3364. 12521995 10.1182/blood-2002-10-3071

[22] Richardson DK, Phibbs CS, Gray JE, et al. (1993) Birth weight and illness severity: independent predictors of neonatal mortality. Pediatrics 91, 969–975. 8474818

[23] Lawn JE, Cousens S, Bhutta ZA, et al. (2004) Why are 4 million newborn babies dying each year? Lancet 364, 399–401. 15288723 10.1016/S0140-6736(04)16783-4

[24] Gluckman P and Hanson M (editors) (2006) Developmental Origins of Health and Disease. Cambridge: University Press.

[25] Barker DJ (1995) Fetal origins of coronary heart disease. BMJ 311, 171–174. 7613432 10.1136/bmj.311.6998.171 PMC2550226

[26] Bhutta AT, Cleves MA, Casey PH, et al. (2002) Cognitive and behavioral outcomes of school-aged children who were born preterm: a meta-analysis. JAMA 288, 728–737. 12169077 10.1001/jama.288.6.728

[27] Gardosi J (2004) Customised fetal growth standards: rationale and clinical application. Semin Perinatol 28, 33–40. 15058900 10.1053/j.semperi.2003.12.002

[28] National Institute for Clinical Excellence (NICE) (2008) Antenatal Care: Routine Care for the Healthy Pregnant Woman. London: NICE.

[29] Schluchter MD (2008) Flexible approaches to computing mediated effects in generalized linear models: generalized estimating equations and bootstrapping. Multivar Behav Res 43, 268–288. 10.1080/00273170802034877 26765663

[30] Alwan NA, Greenwood DC, Simpson NA, et al. (2010) Iron intake during early pregnancy and birth size: insights revealed through structural equation modelling. Proc Nutr Soc 69, E592.

[31] Alwan NA, Greenwood DC, Simpson NA, et al. (2010) The relationship between dietary supplement use in late pregnancy and birth outcomes: a cohort study in British women. BJOG 117, 821–829. 20353456 10.1111/j.1471-0528.2010.02549.x PMC2874518

[32] Bergmann RL, Gravens-Müller L, Hertwig K, et al. (2002) Iron deficiency is prevalent in a sample of pregnant women at delivery in Germany. Eur J Obstet Gynecol Reprod Biol 102, 155–160. 11950483 10.1016/s0301-2115(01)00609-1

[33] Beard JL (1994) Iron deficiency: assessment during pregnancy and its importance in pregnant adolescents. Am J Clin Nutr 59, 502S–508S. 8304288 10.1093/ajcn/59.2.502S

[34] Howie G, Sloboda D, Kamal T, et al. (2009) Maternal nutritional history predicts obesity in adult offspring independent of postnatal diet. J Physiol 587, 905–915. 19103681 10.1113/jphysiol.2008.163477 PMC2669979

[35] Scholl TO & Hediger ML (1994) Anemia and iron-deficiency anemia: compilation of data on pregnancy outcome. Am J Clin Nutr 59, 492S–500S (discussion 500S–501S). 8304287 10.1093/ajcn/59.2.492S

[36] Brion M, Leary S, Davey Smith G, et al. (2008) Maternal anemia, iron intake in pregnancy, and offspring blood pressure in the Avon Longitudinal Study of Parents and Children. Am J Clin Nutr 88, 1126–1133. 18842803 10.1093/ajcn/88.4.1126

[37] Alwan NA, Greenwood DC, Simpson NA, et al. (2011) Dietary iron intake during early pregnancy and birth outcomes in a cohort of British women. Hum Reprod 26, 911–919. 21303776 10.1093/humrep/der005 PMC3057752

[38] Scanlon KS, Yip R, Schieve LA, et al. (2000) High and low hemoglobin levels during pregnancy: differential risks for preterm birth and small for gestational age. Obstet Gynecol 96, 741–748. 11042311 10.1016/s0029-7844(00)00982-0

[39] Scholl TO, Hediger ML, Fischer RL, et al. (1992) Anemia *vs* iron deficiency: increased risk of preterm delivery in a prospective study. Am J Clin Nutr 55, 985–988. 1570808 10.1093/ajcn/55.5.985

[40] James WPT, Norum K, Smitasiri S, et al. (2000) Ending malnutrition by 2020: an agenda for change in the millennium. Final report to the ACC/SCN by the Commission on the Nutrition Challenges of the 21st Century. Food Nutr Bull 21, 1–88.

[41] Rasmussen KM (2001) Is there a causal relationship between iron deficiency or iron-deficiency anemia and weight at birth, length of gestation and perinatal mortality? J Nutr 131, 590S–603S. 11160592 10.1093/jn/131.2.590S

[42] Ribot B, Aranda N, Viteri F, et al. (2012) Depleted iron stores without anaemia early in pregnancy carries increased risk of lower birthweight even when supplemented daily with moderate iron. Hum Reprod 27, 1260–1266. 22357769 10.1093/humrep/des026

[43] Remacle C, Bieswal F, Bol V, et al. (2011) Developmental programming of adult obesity and cardiovascular disease in rodents by maternal nutrition imbalance. Am J Clin Nutr 94, 1846S–1852S. 21543546 10.3945/ajcn.110.001651

[44] Royal College of Obstetricians and Gynaecologists (2013) Small-for-Gestational-Age Fetus, Investigation and Management (Green-top Guideline No. 31). London: Royal College of Obstetricians and Gynaecologists.

[45] Ruivard M, Boursiac M, Mareynat G, et al. (2000) Diagnosis of iron deficiency: evaluation of the ‘soluble transferrin receptor/transferrin’ ratio. Am J Clin Nutr 21, 837–843 (in French). 10.1016/s0248-8663(00)00234-4 11075392

[46] Cook JD (2005) Diagnosis and management of iron-deficiency anaemia. Best Pract Res Clin Haematol 18, 319–332. 15737893 10.1016/j.beha.2004.08.022

